# A Patient With Idiopathic Recurrent Acute Pancreatitis Demystified by Endoscopic Ultrasound

**DOI:** 10.7759/cureus.57758

**Published:** 2024-04-07

**Authors:** Rahul Samanta, Prasanta Debnath, Pradeepta Sethy, Habung Mobing, Ramanand Modak

**Affiliations:** 1 Gastroenterology, Medica Superspecialty Hospital, Kolkata, IND

**Keywords:** complication, hepatobiliary tree, endoscopic ultrasound, recurrent acute pancreatitis, ascaris lumbricoides

## Abstract

Ascariasis is a common helminthic infection, especially in India. Though it is known to inhabit the jejunum of the small intestine, it may invade the hepato-biliary and pancreatic ducts, causing a spectrum of clinical manifestations. We present a case of idiopathic recurrent acute pancreatitis in a 61-year-old female patient who was later diagnosed with pancreatic ascariasis using endoscopic ultrasound. This emphasizes the possibility of ascariasis as one of the causes of acute pancreatitis and the use of endoscopic ultrasound as an initial diagnostic tool for the evaluation of idiopathic acute pancreatitis.

## Introduction

Recurrent acute pancreatitis is defined as the occurrence of two or more episodes of acute pancreatitis without underlying chronic pancreatitis. Despite thorough evaluation, no definite etiology is identified in 10-30% of these patients, hence they are labeled as idiopathic recurrent acute pancreatitis [1]. Ascaris lumbricoides, a helminth, infects 70% of the population in endemic countries like India. This worm typically resides in the jejunum but may migrate proximally to enter the hepato-biliary and pancreatic ducts through the ampulla of Vater. Patients may present with symptoms such as biliary colic, acute cholangitis, acalculous cholecystitis, hepatic abscess, acute pancreatitis, and hepaticolithiasis [2].

## Case presentation

**Figure 1:**
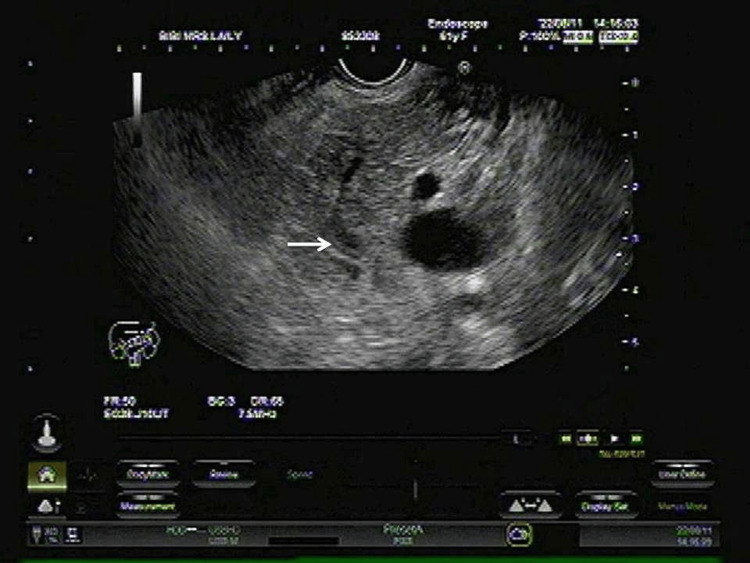
Linear endoscopic ultrasound showing a long, linear, hyper-echogenic structure without acoustic shadowing in the main pancreatic duct (white arrow).

We report a case of a 61-year-old woman from a remote Indian village, of low socioeconomic status, who presented with complaints of abdominal pain associated with nausea and vomiting for two days. She had a similar episode two months prior, for which she was admitted to a local hospital and diagnosed and treated for acute pancreatitis. No definite etiology was identified at that time. During the current admission, a detailed history was taken. She reported no history of fever, weight loss, alcohol intake, gallstone disease, drug intake, abdominal trauma, recent surgery, or endoscopic procedures. She was non-diabetic and had no history of dyslipidemia. Blood reports showed significant elevation of amylase and lipase levels. Complete hemogram, liver function tests, calcium, and triglyceride levels were all within normal ranges. An abdominal ultrasound revealed a bulky pancreas with mild hepatomegaly. An endoscopic ultrasound (EUS) was performed using a linear echoendoscope (Pentax EG 3830 UT, Japan) with a Hitachi Avius processor at a 7.5 MHz frequency. It showed a heterogeneous echotexture of the pancreas with multiple hypoechoic areas in the head, body, and tail of the pancreas. The main pancreatic duct (MPD) was dilated, measuring 4 mm in the head region. A hyperechoic linear lesion without acoustic shadowing was observed in the MPD from the genu to the mid-body of the pancreas, suggestive of a roundworm (Figure 1).

**Figure 2:**
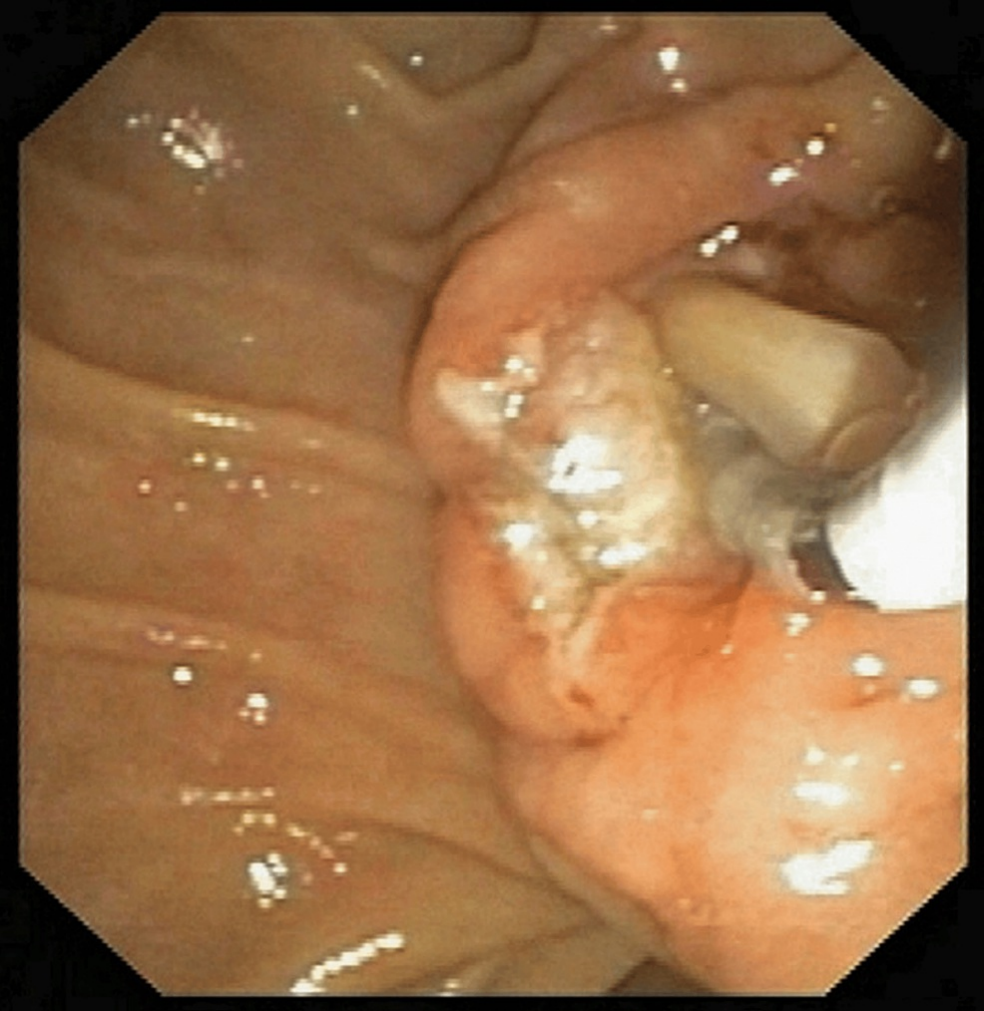
Endoscopic retrograde cholangiopancreatography (ERCP) was performed and balloon sweeps were taken to remove the worm.

**Figure 3:**
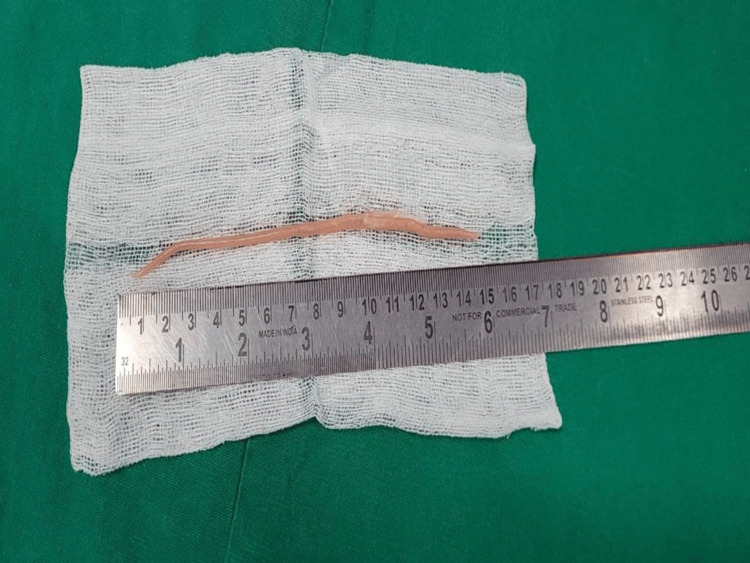
A single Ascaris worm measuring 15 cm extracted out of the pancreatic duct by endoscopic retrograde cholangiopancreatography (ERCP).

Endoscopic retrograde cholangiopancreatography (ERCP) was performed the same day, which showed a dilated MPD with dilated side branches and a long linear defect suggestive of a worm. Multiple balloon sweeps were performed, and a single Ascaris worm measuring 15 cm was removed (Figures 2, 3).

Following the procedure, the patient experienced significant clinical improvement. A stat dose of tablet Albendazole 400 mg was administered. The patient was reviewed again at one month, and she had a complete resolution of symptoms. A repeat EUS was performed, which showed a normal pancreas and pancreatic duct. Subsequently, the patient was followed up on a monthly basis for the next six months. She remained asymptomatic, and there was no recurrence of pancreatitis.

## Discussion

*Ascaris lumbricoides* is a common intestinal parasite worldwide, infecting approximately one-fourth of the global population. These helminths may migrate and invade the biliary and pancreatic ducts. Depending on the site of invasion, patients may present with symptoms ranging from biliary colic and acute cholangitis to acute cholecystitis, acute pancreatitis, and, in rare cases, hepatic abscess or hemobilia [3].

Laboratory investigations often do not aid in the diagnosis of hepato-biliary and pancreatic ascariasis [2]. Abdominal ultrasound (US) serves as an efficient, reliable, and non-invasive diagnostic tool for identifying hepato-biliary, enteric, and pancreatic ascariasis [4]. However, it has limitations in detecting single worms, duodenal ascariasis, worms invading the ampullary orifice, and pancreatic worms [2,5]. Other diagnostic modalities for evaluating hepato-biliary and pancreatic ascariasis include EUS, CT, and MRI. EUS is the preferred method for assessing the biliary tree and pancreas in cases of suspected pancreato-biliary ascariasis, where *Ascaris lumbricoides* appears as a long echogenic structure with a central anechoic linear defect, without any shadow effect [6].

The treatment options for biliary ascariasis range from non-surgical and surgical to endoscopic approaches. Non-surgical treatment includes the use of antihelminthics, antispasmodics, and analgesics. In cases of poor medical response, surgical intervention may be considered. ERCP is effective for both diagnosis and treatment, achieving high success rates in the removal of worms from the biliary tree [7]. Preventive measures include the enhancement of sanitation, personal hygiene, health education, and the implementation of deworming programs [2].

Therefore, ascariasis should be considered as a possible cause of acute pancreatitis, especially in regions where the prevalence of ascariasis is high or sanitation and hygiene are poor [8]. Few cases of pancreatic ascariasis have also been reported from non-endemic countries [9,10]. EUS can be considered the first-line investigation for etiological evaluation of idiopathic acute pancreatitis [1].

## Conclusions

*Ascaris lumbricoides* can cause recurrent acute pancreatitis by invading the hepato-biliary and pancreatic ducts and should be considered as one of the etiologies of recurrent idiopathic acute pancreatitis. EUS is an efficient tool for the evaluation of idiopathic acute pancreatitis.
